# Is It Just?

**Published:** 1897-04

**Authors:** 


### IS IT JUST?

Upon another page of this number will be found a severe criticism of the New York Dental College, quoted in part from the New York *Tribune.* The college may or may not deserve the castigation given by the president of the Massachusetts Dental Society, but the impression left upon the minds of readers will be that this college is a State institution. The understanding has always been that it was founded upon a charter granted by the State of New York, and supported by private funds. It is presumed its organization partook somewhat of the character of a partnership concern, the faculty furnishing the means to sustain it. If this be so, it seems hardly proper to complain that the faculty have done a great wrong, that after expending all required on the students' training they divided the surplus. The question of amount is not to be considered; for if it be wrong to divide forty-five thousand, it would be equally wrong to divide forty-five hundred, or any lesser amount. It is simply a matter of legitimate business, and might with equal propriety apply as well to the prosperous dentist who invests his surplus yearly.

Whether colleges are right or wrong in being thus organized is not the question at issue; but had dental education waited until States, universities, and medical colleges were ready to proffer the means necessary to sustain them, it is safe to assume that dental training would be to-day where it was in 1850. The history of the financial sacrifices made by dental faculties to educate the dental profession to a higher standard of training, as well as that of the public, has never been written, and probably will never be appreciated.

It is presumed the faculty of the New York College of Dentistry can speak for itself, if so disposed; but in our judgment the arraignment of a school, presumably owned by the faculty, for dividing the profits as their remuneration, is on its face, and in the absence of more definite information, very unjust.

